# Laryngeal airway reconstruction indicates that rodent ultrasonic vocalizations are produced by an edge-tone mechanism

**DOI:** 10.1098/rsos.170976

**Published:** 2017-11-01

**Authors:** Tobias Riede, Heather L. Borgard, Bret Pasch

**Affiliations:** 1 Department of Physiology, Midwestern University, 19555 N 59th Avenue, Glendale, AZ 85308, USA; 2 Department of Biological Sciences, Northern Arizona University, 617 S. Beaver Street, Flagstaff, AZ 86011, USA; 3 Merriam-Powell Center for Environmental Research, Northern Arizona University, Flagstaff, AZ 86011, USA; 4 Center for Bioengineering Innovation, Northern Arizona University, Flagstaff, AZ 86011, USA

**Keywords:** bioacoustics, vocal production, evolution, acoustic communication, source-filter theory

## Abstract

Some rodents produce ultrasonic vocalizations (USVs) for social communication using an aerodynamic whistle, a unique vocal production mechanism not found in other animals. The functional anatomy and evolution of this sound production mechanism remains unclear. Using laryngeal airway reconstruction, we identified anatomical specializations critical for USV production. A robust laryngeal cartilaginous framework supports a narrow supraglottal airway. An intralaryngeal airsac-like cavity termed the ventral pouch was present in three muroid rodents (suborder Myomorpha), but was absent in a heteromyid rodent (suborder Castorimorpha) that produces a limited vocal repertoire and no documented USVs. Small lesions to the ventral pouch in laboratory rats caused dramatic changes in USV production, supporting the hypothesis that an interaction between a glottal exit jet and the alar edge generates ultrasonic signals in rodents. The resulting undulating airflow around the alar edge interacts with the resonance of the ventral pouch, which may function as a Helmholtz resonator. The proposed edge-tone mechanism requires control of intrinsic laryngeal muscles and sets the foundation for acoustic variation and diversification among rodents. Our work highlights the importance of anatomical innovations in the evolution of animal sound production mechanisms.

## 1. Introduction

Rodents employ diverse vocal behaviour in a variety of social contexts [1–6]. Rats and mice produce both audible and ultrasonic vocalizations (USVs) [7], with fundamental frequencies between 100 and 120 000 Hz. Ultrasonic whistling is the most notable behaviour and the only clear example of sound production by a whistle mechanism among vertebrates, besides human whistling [8]. Although such a whistle mechanism has been suspected in birds, frogs, dolphins and non-human primates [9–13], it has been convincingly demonstrated using heliox experiments only in laboratory mice [14], rats [15] and grasshopper mice [16]. Abolishment of USVs following interruption of the nerve supply to laryngeal muscles [14,17,18] and USV generation from excised larynges [19–21] indicate that the larynx is the site of sound production. However, understanding the evolution and physiological mechanisms of how aerodynamic energy is converted into sound remains controversial and often detached from anatomical detail.

The morphology of the mammalian larynx has received considerable attention [22–24]. The complex structure consists of a cartilaginous framework and at least five pairs of intrinsic muscles. However, although rodents represent more than 40% of mammalian diversity [25], few detailed morphological descriptions of the rodent larynx exist [26–28], in part due to challenges associated with its small size. Roberts [14] suggested that the mouse larynx shows ‘no modifications for ultrasound production'. Roberts’ hypothesis posited that glottal airflow impinges on a downstream hole (hole-tone whistle hypothesis) [29–32], yet the identity of this structure has not been determined. A recent alternative hypothesis suggests that glottal airflow impinges as a high-speed jet on a planar surface purportedly provided by the thyroid cartilage [20]. However, the intralaryngeal supraglottal space is narrow and has a small ventral opening into a larger spherical cavity termed the ventral pouch, a structure not considered by Roberts [14] and Mahrt *et al*. [20] but previously described (e.g. [23,26,33–35]). A planar surface does not physically exist, and the function of the ventral pouch has not been addressed.

The mechanism of energy conversion depends critically on the complex geometry of the upper respiratory tract which has important consequences for glottal flow [36]. The airflow passing the glottis (glottal jet) interacts with supraglottal morphologies, producing complex vortex structures [37]. The geometry of the laryngeal airway is therefore critical for understanding audible and ultrasonic vocal production in rodents.

To elucidate how the rodent larynx is adapted for USVs, we conducted a comparative anatomical investigation in three muroid rodents that commonly produce USVs and one outgroup that does not. Recently available micro-computed tomographic (CT) imaging in conjunction with tissue staining provides relevant three-dimensional insight into small, complex anatomical structures [38,39]. We used contrast-enhanced microCT imaging and traditional histology to investigate the larynges of laboratory mice (*Mus musculus*), grasshopper mice (genus *Onychomys*), laboratory rats (*Rattus norvegicus*) and kangaroo rats (*Dipodomys ordii*). Kangaroo rats (suborder Castorimorpha, family Heteromyidae) have a limited vocal repertoire and no documentation of USVs [40–44] and therefore served as behavioural outgroup. We also performed an experimental manipulation to investigate the functional role of an intralaryngeal airsac-like structure, the ventral pouch, in USV production.

## 2. Material and methods

### 2.1. Animals

Investigations were performed on six grasshopper mice (two *O. arenicola*, one *O. torridus*, three *O. leucogaster* and an F1 hybrid between *O. arenicola* and *O. torridus*), six laboratory mice (*M. musculus*, CD1 strain; four males and two females), 12 laboratory rats (*R. norvegicus*, Sprague–Dawley; eight males and four females) and four kangaroo rats (*D. ordii*; two males and two females). The animals were deeply anaesthetized with xylazine (8 mg kg^−1^) and ketamine (80 mg kg^−1^), cardially perfused with saline solution and subsequently with paraformaldehyde. Larynges were dissected and fixed in 10% buffered formalin phosphate (SF100-4; Fisher Scientific) for 24 h. All specimens were processed at Midwestern University, Glendale, AZ.

### 2.2. Laryngeal airway reconstruction: micro-computed tomographic imaging and histology

The larynges of four grasshopper mice, four laboratory mice, four laboratory rats and four kangaroo rats (two males and two females from each species) were X-rayed. Iodine staining protocols modified from previous studies [38,39] were used. Each specimen was transferred into 70% ethanol for 2 days, then into pure ethanol (greater than 99.5%) for 3 days and then into 0.2% (0.4 g iodine/200 ml 99% ethanol) or 1% iodine-based ethanol solution (2 g iodine/200 ml 99% ethanol) for staining (electronic supplementary material, table S1). After 3 days, the staining solution was renewed and the tissue was stained for another 10–14 days. After staining, the specimen was placed in a custom-made acrylic tube and scanned in air. Scanning was done using a Skyscan 1172 (Bruker). Reconstructed image stacks were then imported into AVIZO software (v. Lite 9.0.1). Larynx cartilages and the border between the airway and soft tissues of the larynx in the CT scans were traced manually. This approach provided outlines of the cartilaginous framework and the airway (electronic supplementary material, videos S1--S4).

The larynges of two grasshopper mice, two laboratory mice and two laboratory rats (two males from each species) were histologically processed. One specimen from each species was sectioned in the coronal plane and the other in the sagittal plane. Sections (5 µm thick) were collected every 50 µm. Sections were stained with haematoxylin and eosin for a general overview, and scanned with ImageScope software (v. 8.2.5.1263; Aperio Tech.).

### 2.3. Surgical manipulation of the ventral pouch

We addressed the hypothesis that the ventral pouch plays a functional role in ultrasonic whistle production with six rats (four males and two females; Sprague–Dawley). The animal was anaesthetized (xylazine, 8 mg kg^−1^ and ketamine, 80 mg kg^−1^). The glottis and ventral pouch were visualized by an oral approach. A cautery was used to lesion the ventral pouch. Great care was taken to not compromise the vocal folds. The presence of normal respiratory vocal fold movements and uninjured vocal folds was confirmed visually in each animal at the conclusion of the surgery. Operated rats received fluids (Ringer, 10 ml sc), antibiotics (Baytril 20 mg kg^−1^ orally) and analgesics (buprenorphine, 0.3 mg kg^−1^ sc) following surgery to promote recovery.

Each animal was acoustically monitored three times for 24 h: once prior to the surgery, once after waking up from surgery, and a third time two weeks after surgery. Recordings were done with an ultrasonic microphone (Avisoft Bioacoustics, CM16/CMPA-5 V; placed 5 cm over the centre of the cage). Sound was analysed for the presence of 22 and 50 kHz USVs. During the recording sessions, an animal was stimulated with olfactory cues from other animals (bedding) in order to induce 50 kHz calls and with facial air puffs to trigger 22 kHz calls. Animals were sacrificed immediately following the third recording. The larynx was formalin-fixed, iodine-stained and submitted to microCT imaging in order to evaluate the extent of the alteration of the laryngeal airway.

## 3. Results

### 3.1. Cartilaginous framework of the larynx

In all four species, the cartilaginous framework of the larynx consists of thyroid cartilage, epiglottis, a pair of arytenoid cartilages and cricoid cartilage (electronic supplementary material, videos S1--S4). The three muroid species also possess an alar cartilage that was not present in *Dipodomys*.

**Figure 1.**
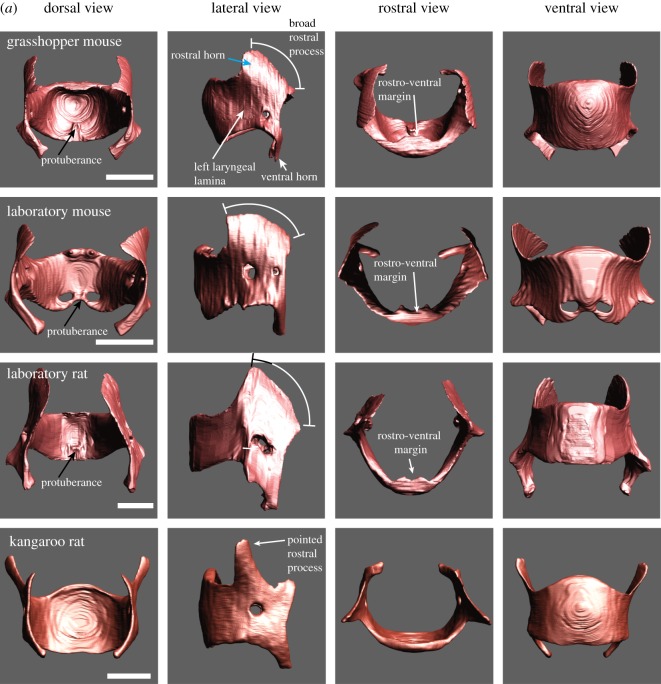

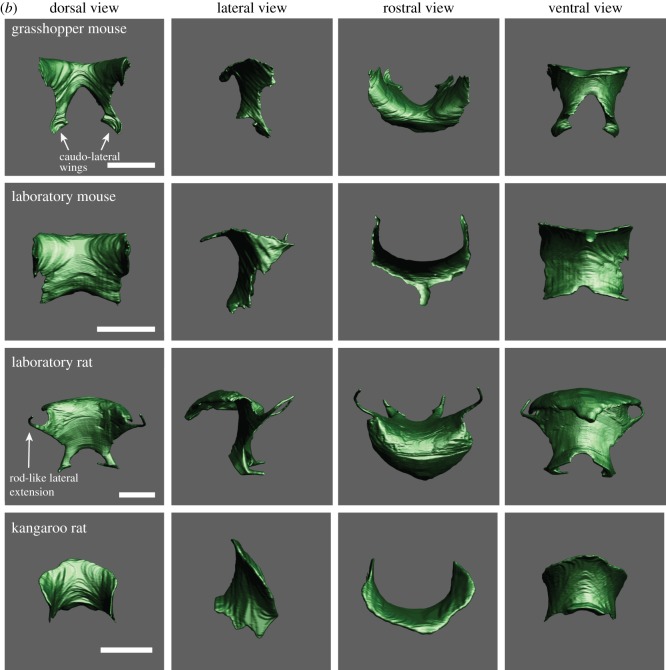

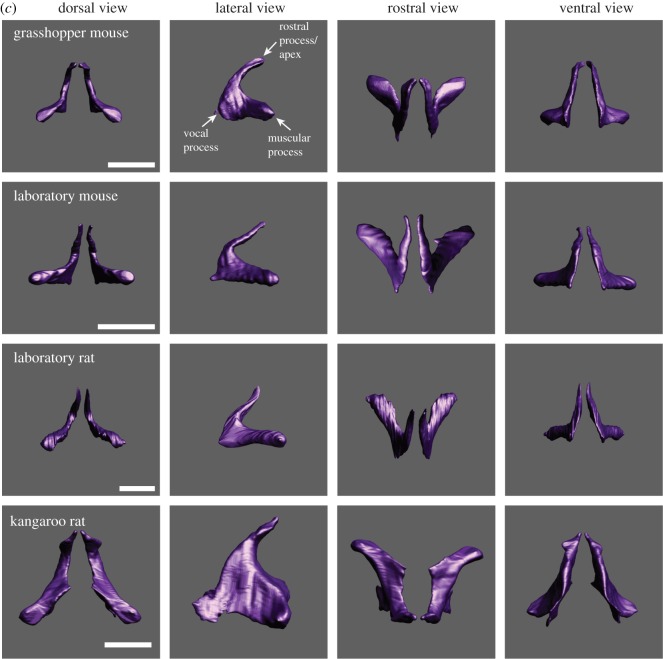

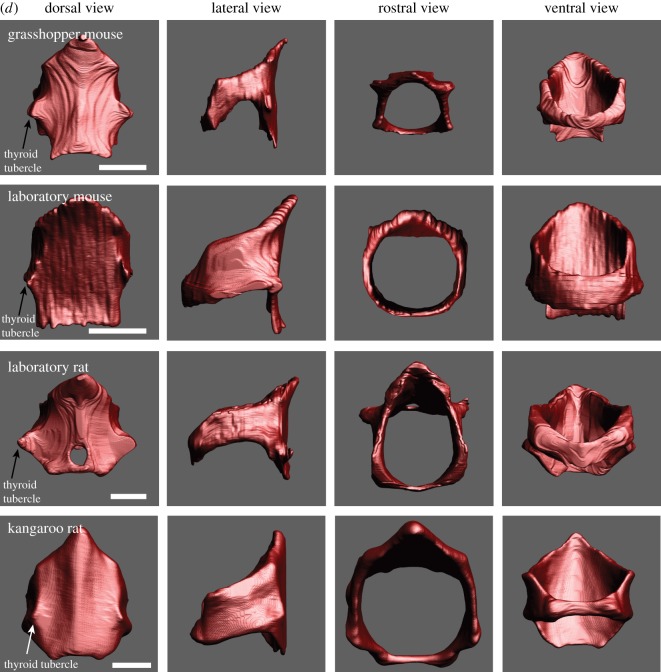
(*a*) The laryngeal cartilaginous skeletons in four rodent species show features that support and reinforce the supraglottal airway. A bent rostro-ventral margin of the thyroid cartilage was present in all four species. A highly mineralized protuberance on the medial surface of the thyroid cartilage was found in *Onychomys*, *Mus* and *Rattus*. The protuberance serves as anchor for the vocal folds. One or two holes in the lateral laminae (foramen thyroideum) were found in all four species. Laboratory mice have an additional pair of foraminae to the left and right of the protuberance. The rostral process is broad in the three muroid species, but pointed and narrow in kangaroo rats. Reference bar is 1 mm. STL files for all cartilages and the airway can be viewed on Morphobank. (*b*) One representative example of the epiglottis from each species. Large caudo-lateral wings are present in grasshopper mice and rats. Rats also possess thin rod-like lateral extensions that support the aryepiglottic fold. Reference bar is 1 mm. (*c*) One representative pair of arytenoid cartilages from each species. Reference bar is 1 mm. (*d*) One representative example of the cricoid cartilage from each species. A hole in the dorsal plate (foramen cricoideum) was only found in rats. Reference bar is 1 mm.

The thyroid cartilage consists of a left and right lamina each with a rostral and caudal horn (figure 1*a*). The rostral horn is broad in *Mus*, *Rattus* and *Onychomys,* but smaller and pointed in *Dipodomys*. The rostro-ventral margin of the thyroid bends dorsally in all four species. The specific shape of the rostro-ventral margin in rodents had been recognized by Harrison [24] as an ‘anterior bowing of the thyroid’ and by Schneider [23] who noted that the cranial edge of the thyroid cartilage is bent towards the laryngeal lumen forming a bulla thyroidea. In the three muroid species, a ventral pouch is embedded in this bulla. The ventral pouch is explained in more detail in the next section. The supraglottal intralaryngeal space is much broader and does not contain a ventral pouch in *Dipodomys*.

In all three muroid species, a small protuberance is present on the medial surface of the thyroid cartilage in a caudal mid-sagittal position (figure 1*a*). The structure consists of highly mineralized cartilage that protrudes 200 µm into the laryngeal lumen. Vocal folds attach to the thyroid cartilage via this protuberance. The protuberance is also part of the caudal wall of the ventral pouch. It contributes to the robust support of the ventral pouch. The protuberance is absent in *Dipodomys*.

In all three muroid species, the epiglottis is relatively large (figure 1*b*). The epiglottis consists of a ventral plate. Lewis & Prentice [45] had recognized long caudo-lateral wings of the epiglottis in rats. In both grasshopper mice and rats, such large wings are present. The wings surround the supraglottal cavity on both sides, including the ventral pouch. Rats also possess thin extensions on each side which support the aryepiglottic fold. The epiglottis slides around the cranio-ventral surface of the rostro-ventral margin of the thyroid cartilage in order to open or close the laryngeal lumen (figure 1*b*).

The arytenoid cartilages consist of a vocal process and a short muscular process (figure 1*c*). The vocal process is part of the glottis. The mammalian glottis consists of a membranous portion (soft tissue including epithelium, lamina propria and thyroarytenoid muscle) and a cartilaginous portion (a vocal process of the arytenoid cartilage). The relative lengths between these two portions affect the location of the glottal opening during sound production and therefore the position and direction of the glottal airflow. In all four rodents, the cartilaginous section measures about 0.8–1 mm and the membranous portion 0.5 mm.

The cricoid cartilage (figure 1*d*) forms a complete ring with a broad plate dorsally and a narrow band laterally and ventrally. In laboratory rats and grasshopper mice, the thyroid tubercles, part of the thyro-cricoid joint, are very prominent (figure 1*d*). Furthermore, rats have a foramen in the dorsal lamina.

**Figure 2.**
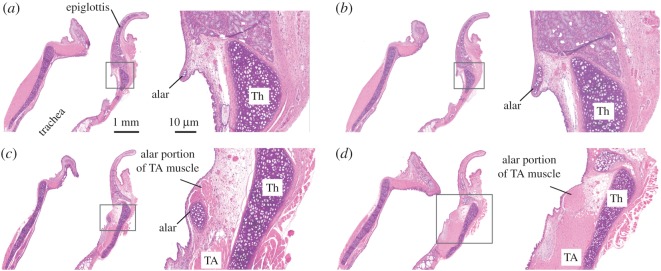
Histological sagittal sections of the larynx of a grasshopper mouse (haematoxylin and eosin stain). Sections are mid-sagittal (*a*) and more lateral (*b–d*). The location of the high magnification image on the right is indicated in the inset in the left image of each panel. Reference bars in (*a*) apply to all panels. Note that the endpoint of the alar cartilage in (*c*) and (*d*) is associated with muscular tissue which represents a branch of the thyroarytenoid muscle. TA, thyroarytenoid muscle; Th, thyroid cartilage.

An alar cartilage is present in muroid rodents but not in kangaroo rats. The alar cartilage is well known in rats and mice (e.g. [26,27,46,47]). It is a very small cigar- or half-ring-shaped structure and is located in the rostral edge of the entrance to the ventral pouch (figure 2). A small portion of the thyroarytenoid muscle extends towards and attaches to the endpoints of the alar cartilages (figure 2).

### 3.2. The intralaryngeal airway and ventral pouch

**Figure 3.**
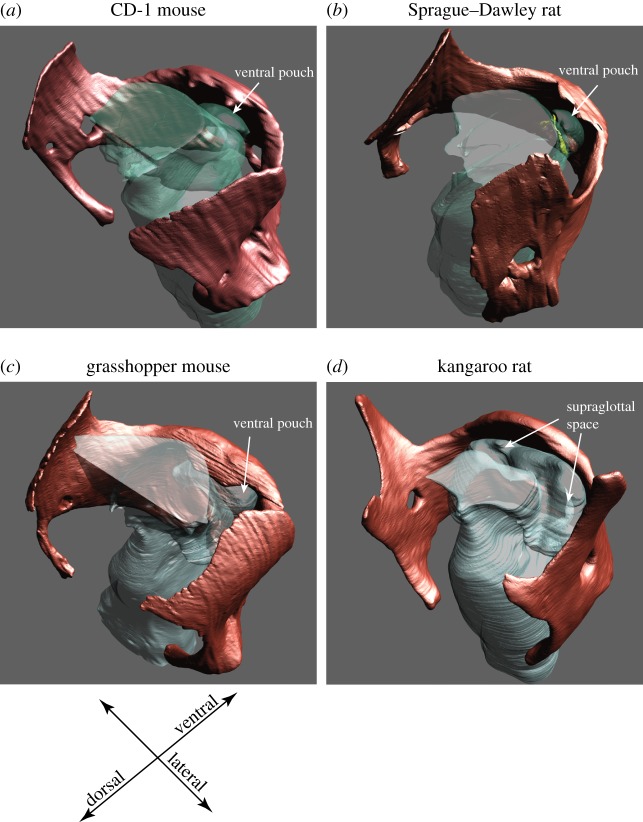
The supraglottal intralaryngeal airway is narrow in laboratory mice, rats and grasshopper mice. The broad rostral process of the thyroid cartilage and the bent rostro-ventral margin contribute to the confinement of the intralaryngeal lumen (including the ventral pouch) in the three muroid rodents (*a–c*) but not in the kangaroo rat (*d*).

The laryngeal airway plays a critical role in vocal production. The supraglottal intralaryngeal space is tightly constricted in muroid rodents (figure 3). The bent rostro-ventral margin and the broad rostral process of the thyroid confine the lumen to a much smaller area than in *Dipodomys*.

**Figure 4.**
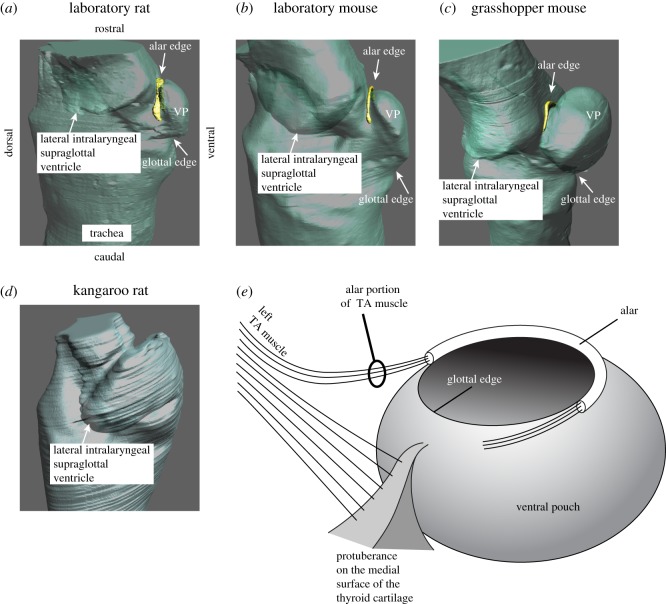
The alar cartilage (yellow) is embedded in the rostral edge (alar edge) of the entrance to the ventral pouch in *Rattus* (*a*), *Mus* (*b*) and *Onychomys* (*c*) but not in *Dipodomys* (*d*). Note that the intralaryngeal supraglottal ventricle is much broader in *Dipodomys* than the ventral pouch in the muroid species. (*e*) Schematic of the ventral pouch indicating the position of the alar cartilage. Fibres of the thyroarytenoid muscle (=alar portion of the TA muscle) insert on the endpoints of the alar cartilage. A contraction would reduce the size of the entrance to change the shape of the ventral pouch. VP, ventral pouch; TA, thyroarytenoid.

A ventral pouch had been identified in Muridae and Arvicola [23] and is present early in embryological development [33]. Schneider [23] hypothesized that the ventral pouch in rodents is a primitive version of a ventral laryngeal air sac present in many non-human primates. The ventral pouch is positioned cranially (i.e. downstream from the vocal folds) and is surrounded by four cartilaginous structures, including (i) the cartilaginous wings of the epiglottis, (ii) the bent rostro-ventral margin of the thyroid cartilage and (iii) the protuberance on the medial surface of the thyroid cartilage. Part of the edge of its entrance is re-enforced by the alar cartilage (iv). Hereafter, we operationally define the portion of the entrance hole containing the alar cartilage as the ‘alar edge’ (figure 4*a–c*) and the opposite site closer to the glottis as the ‘glottal edge’ of the entrance to the ventral pouch. The glottal edge is supported by the protuberance on the medial surface of the thyroid cartilage (figure 4).

Kangaroo rats do not possess a ventral pouch or an alar cartilage like the muroid species (figures 3*d* and 4*d*). Instead, the supraglottal space is wide and extends laterally within the laryngeal confinements.

The presence of the alar cartilage accomplishes two functions. First, it reinforces the entrance of the ventral pouch. Second, it serves as a shield that reduces the opening of the ventral pouch as contraction of the alar portion of the thyroarytenoid muscle pulls the alar edge caudally (figure 4*e*).

**Figure 5.**
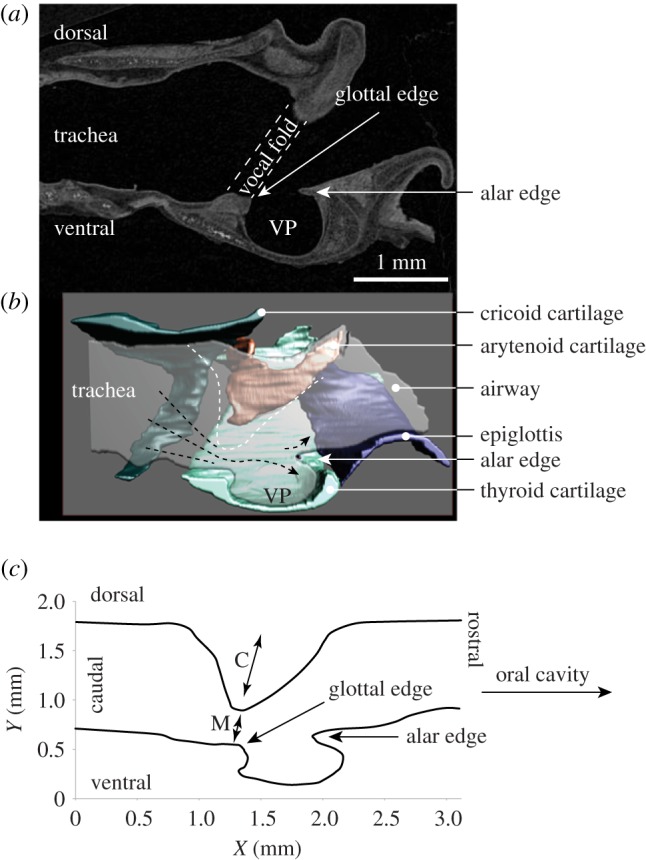
Sagittal section of a grasshopper mouse larynx. (*a*) Iodine-stained computer tomographic image. (*b*) Three-dimensional model of the cartilages and the intralaryngeal airway. (*c*) Mid-sagittal dimension of the airway of a grasshopper mouse. The white dashed line indicates the dorsal boundary of the airway as it becomes constricted coincident with vocal fold abduction. The glottal opening is close to the ventral surface, with the glottal jet flowing into the supraglottal (epilaryngeal) space to pass over the ventral pouch and impinge upon the alar edge. VP, ventral pouch; M, membraneous portion of the vocal fold; C, cartilaginous portion (supported by vocal process of the arytenoid cartilage) of the vocal fold.

We used airway reconstruction to illustrate airflow through the glottis and into the supraglottal space. Figure 5*a* shows the mid-sagittal image of a grasshopper mouse larynx. Figure 5*b* shows the manually reconstructed intralaryngeal lumen embedded in the laryngeal cartilaginous framework. Glottal airflow must pass over the entrance of the ventral pouch and impinge upon the cartilage re-enforced alar edge situated opposite the glottal exit. Greater contraction of the alar portion of the thyroarytenoid muscle will result in a shorter distance between the glottal and alar edge and a smaller lumen inside the ventral pouch. The dimension of the airway with adducted vocal folds is shown in figure 5*c*.

### 3.3. Surgical lesion of the ventral pouch alters ultrasonic vocal structure

**Figure 6.**
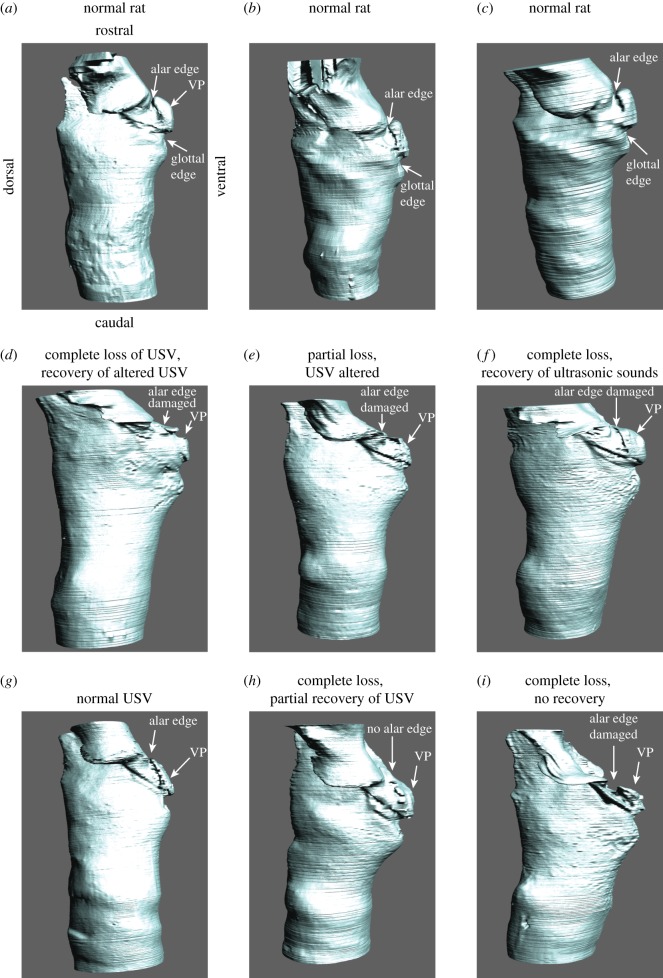
Laryngeal airways of three normal rats (*a–c*) and six rats in which a small lesion targeted the alar edge of the ventral pouch (*d–i*). In six animals (*d–i*), the alar edge, the alar cartilage and the ventral pouch were variably altered to induce complete or partial loss of the normal USV repertoire. In one animal, the alteration of the ventral pouch was minimal (*g*) and no vocal changes were observed. VP, ventral pouch. A description of the lesions in six rats (*d–i*) is provided in table 1. STL files for each airway are archived on Morphobank.

Finally, in order to investigate the function of the ventral pouch in ultrasonic whistle production, we surgically lesioned the ventral pouch in six male rats and found that the magnitude of the damage to the alar edge was associated with the degree of vocal change. The exact lesion placement was not uniform due to challenges of visualizing the entire intralaryngeal space under surgical conditions. We therefore sacrificed rats and visualized the cartilaginous framework and the laryngeal airway by microCT imaging at the end of the experiment (figure 6). Then, we compared the degree of alteration of the ventral pouch with the degree of vocal changes.

**Table 1.** Vocal changes following lesion surgery to the alar edge of the ventral pouch. Animals were recorded before surgery and twice (immediately and two weeks) after surgery. The table lists qualitative differences between the spectrographic appearance of USV observed before and after surgery. None of the animals showed signs of respiratory stress. The table also lists the results of the evaluation of the magnitude of the lesion to the ventral pouch (reconstructed laryngeal airway). After the second sound recording (two weeks after surgery), animals were sacrificed. The dissected larynx was fixed and imaged with contrast-enhanced microCT. USV, ultrasonic vocalization.

rat	vocal change	reconstructed laryngeal airway	diagnosis
alar rat 1, male	*Immediately after surgery*: complete loss of USVs after surgery *Two weeks after surgery*: USVs reminiscent of 50 and 22 kHz calls indicate that sounds occupy normal frequency ranges, but duration and characteristic frequency modulation patterns are different. The rat produces a lot of sniffing when excited by female bedding. Sniffs are sometimes associated with ultrasonic components	Alar cartilage is broken Alar edge is discontinuous, right portion is missing Mucosa of ventral pouch is normal	Severe vocal change associated with damage to the ventral pouch
alar rat 2, male	*Immediately after surgery*: rat produces modified USV. 50 and 22 kHz calls contain sidebands and sound amplitude is reduced *Two weeks after surgery*: 50 kHz calls can be recognized, but fundamental frequencies are lower than prior to surgery. 22 kHz calls are shorter than before surgery	Alar cartilage is not broken Alar edge is intact Mucosa of ventral pouch is thickened	Mild vocal change associated with minor alteration of the mucosa inside an intact ventral pouch
alar rat 3, male	*Immediately after surgery*: complete loss of USVs after surgery. The rat produces sniffs when excited by female bedding, which are associated with ultrasonic components *Two weeks after surgery*: only very short or incomplete 22 kHz calls produced, but the animal shows typical slow breathing motions associated with long 22 kHz call production. No 50 kHz calls are produced, although the animal is stimulated when exposed to female bedding	Alar cartilage is broken Alar edge is partly removed Mucosa of ventral pouch is thickened	Severe vocal change associated with damage to ventral pouch
alar rat 4, male	*Immediately after surgery*: rat produces normal USVs *Two weeks after surgery*: rat produces normal USVs	Alar cartilage is intact Alar edge is continuous Mucosa of ventral pouch is thickened	No vocal change associated with minor alteration of the mucosa inside the ventral pouch
alar rat 5, female	*Immediately after surgery*: complete loss of USVs after surgery *Two weeks after surgery*: modified 50 kHz calls, no 22 kHz calls but typical slow breathing motions associated with long 22 kHz call production	Alar cartilage is broken and alar edge is level with the surface of epiglottis Mucosa of ventral pouch is thickened	Severe vocal change associated with damage to ventral pouch
alar rat 6, female	*Immediately after surgery*: complete loss of USVs *Two weeks after surgery*: no USV. Sniffs sometimes contain USV components	Alar cartilage is broken Alar edge is removed Mucosa of ventral pouch is thickened	Severe vocal change associated with damage to ventral pouch

**Figure 7.**
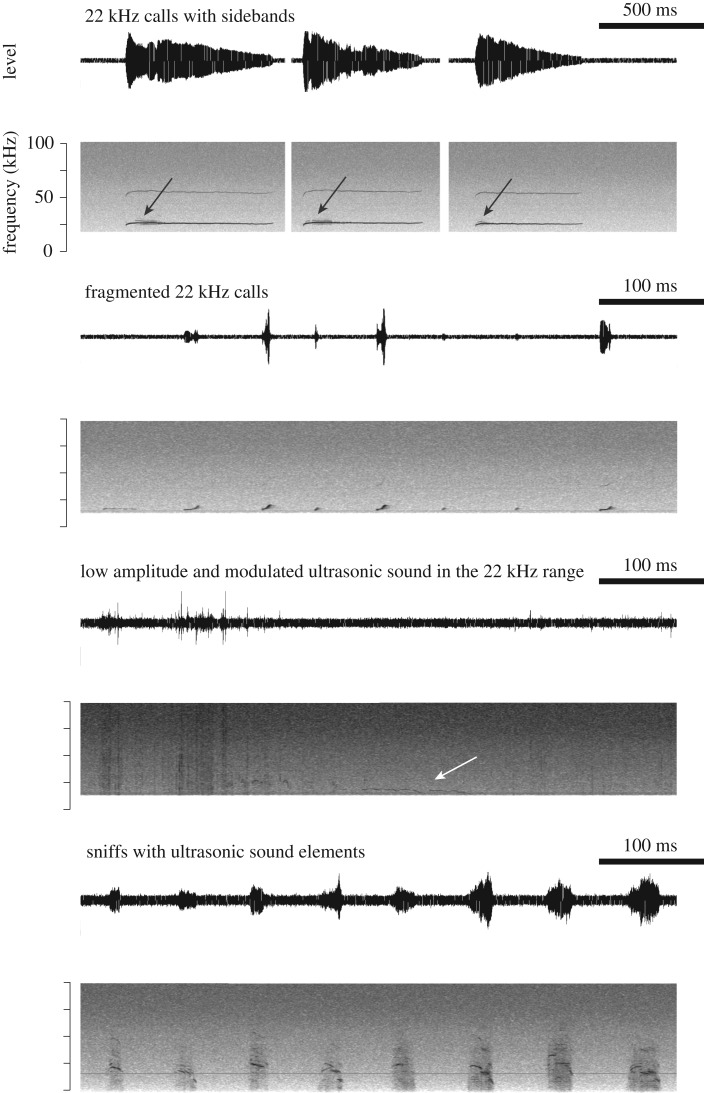
Common vocal changes following lesions to the ventral pouch region of rats*.* In six animals, the alar edge, the alar cartilage and the ventral pouch were variably altered which was associated with complete or partial loss of normal USV repertoire. In one animal, the alteration of the ventral pouch was minimal and no vocal changes were observed.

All six rats produced normal USV before surgery. After surgery, we observed both vocal changes and complete vocal loss. Vocal changes included fragmented 22 kHz calls, 22 kHz calls with sidebands, weak and modulated long calls near 22 kHz, increased rates of sniffing associated with ultrasonic sound production and altered 50 kHz calls (figure 7). Complete vocal loss was inferred from animals exhibiting characteristic locomotor behaviour associated with USV production before surgery [48,49]. For example, 22 kHz USVs were associated with freezing and long exhalations identified by long-lasting slow movements of the thorax and abdomen. Furthermore, we detected no USVs from sexually experienced males that exhibited typical exploratory behaviour associated with 50 kHz calls when exposed to odour from an oestrous female.

One rat produced normal USV after surgery. A second rat produced altered USV but recovered normal USV after two weeks. The four other rats did not produce USV immediately after surgery. Three of the four rats recovered the ability to produce USV two weeks after surgery.

The ventral pouches of six rats showed different degrees of damage ranging from minor thickening of the soft tissue lining of the ventral pouch (one rat with no vocal changes) to the removal of the alar edge and the confinement of the ventral pouch (one rat with complete vocal loss; figure 6). In four animals, the alar edge was variably altered including lesion or removal of the alar cartilage. A summary of morphological and vocal changes is provided in table 1.

## 4. Discussion

Roberts [14,29,30] pioneered the investigation of the functional morphology of rodent laryngeal anatomy and its importance for USV production. In addition to previous work [22–24,26–28,34,47], our findings challenge Roberts' statement that the rodent larynx lacks any specializations. There are at least six structures unique to the larynges of three rodent species that commonly produce ultrasonic whistles. All six features support and reinforce the supraglottal airway. Adaptations include (i) the presence of a ventral pouch supported by (ii) a dorsally bent rostro-ventral margin of the thyroid cartilage; (iii) the wide laterally projecting caudal edges of the epiglottis surrounding the supraglottal chamber, (iv) the alar cartilage, (v) a broad rostral process of the thyroid cartilage and (vi) a highly mineralized protrusion on the medial surface of the thyroid cartilage, which also serves as anchor for the vocal folds. Interestingly, kangaroo rats lacked a number of morphological adaptations associated with USV production. We infer that the absence of such structures is related to investment in an alternative modality of vibratory communication via footdrumming that is unique to the genus [50].

The current study also challenges Roberts’ hole-tone whistle model [29] and the ‘planar impinging model’ of aerodynamic whistle production suggested by Mahrt *et al*. [20]. We discuss our findings in relation to existing proposed mechanisms and present an alternative hypothesis for USV sound production.

### 4.1. How does the laryngeal morphology facilitate aerodynamic whistle production?

#### 4.1.1. Hole-tone whistle model

**Figure 8.**
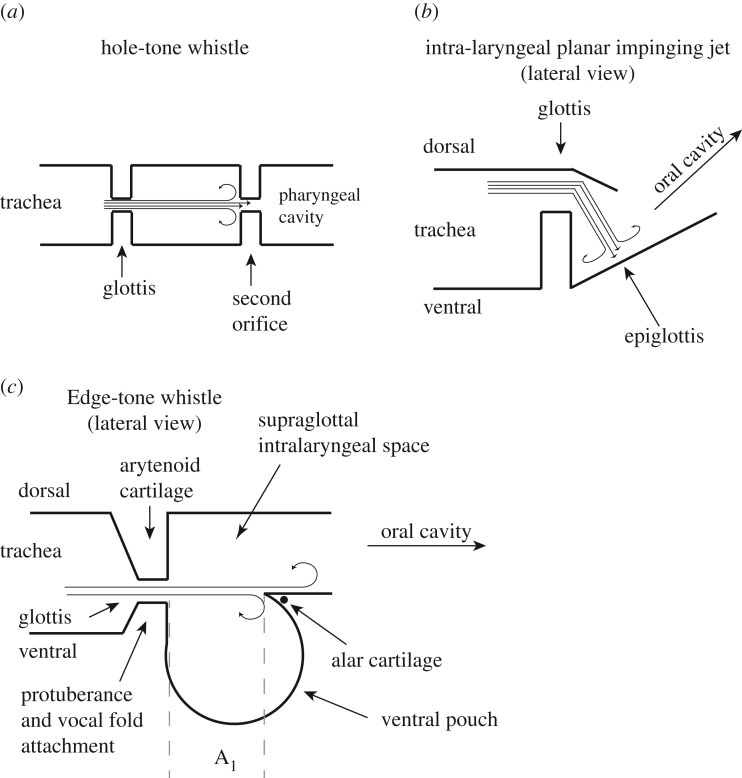
Three models of the whistle production mechanism for USVs in rodents. The hole-tone whistle (*a*) was proposed by Roberts [29]. The identity of the second orifice was not specified. The intralaryngeal planar impinging jet model (*b*) was proposed by Mahrt *et al*. [20]. The location of the glottal exit jet was projected far more dorsally than its actual position, and the role of the ventral pouch had not been specified. The edge-tone model (*c*) suggests that the glottal exit jet is directed at the alar edge. The resulting undulating flow around the alar edge interacts with the resonance of the ventral pouch, which may function as a Helmholtz resonator. The model is based on laryngeal airway reconstruction and takes a rodent-specific laryngeal morphology into account. Fundamental frequency depends on glottal airflow velocity and the glottis-to-edge distance (*A*_1_) [51]. Fundamental frequency (*F*_0_) increases when airflow velocity (*v*) increases and/or when *A*_1_ decreases, according to: *F*_0_ = *v*/2*A*_1_. The Helmholtz resonator would support higher fundamental frequencies if the volume of the ventral pouch decreases simultaneously. Previous studies in *Rattus* demonstrate that electromyographic activity of intrinsic laryngeal muscles, including the thyroarytenoid muscle, is closely associated with frequency changes [32]. The thyroarytenoid muscle indirectly regulates airflow by contributing to vocal fold adduction, and we hypothesize that it is (i) involved in regulating the glottis to alar edge distance and (ii) probably regulates ventral pouch size (figures 2 and 4*e*).

The hole-tone whistle mechanism [29] assumes that the glottal airflow impinges on a second hole located downstream from the glottis (figure 8*a*). Since Roberts' [29] initial study, investigators have only speculated about which anatomical structure might represent this second hole [14,31]. In the current study, we were not able to identify a second valve-like constriction, thus making a hole-tone whistling mechanism highly unlikely. Although Roberts [14] had recognized the ventral pouch (defined as ‘ventricle’) in his anatomical studies of the rodent larynx, he did not assign any vocal function to this unique morphological feature.

#### 4.1.2. The planar impinging jet model

A second model, the planar impinging jet model, was recently proposed [20] based on observations made in the excised laboratory mouse (*Mus*) larynx. The proposed model assumes that glottal airflow impinges perpendicularly on a planar surface. The authors specify ‘…impingement of the coherent flow structures on the planar inner laryngeal wall, consisting of the thyroid, and perhaps partially the epiglottis’ [20] (figure 8*b*). However, it remains unclear which morphological feature represents this planar surface. We were unable to identify such a surface in *Mus* and three other species investigated here. The supraglottal intralaryngeal surface of the thyroid cartilage is part of a complex arrangement of cartilage and soft tissue in which the ventral pouch (not described by Mahrt *et al*. [20]) is embedded and thus does not form a planar surface.

The planar impinging jet model further assumes that airflow is re-routed in order to achieve a perpendicular angulation between the glottal jet exit and the ‘laryngeal inner planar wall’ (figure 8*b*). Airways of the three USV-producing species investigated here indicate that the glottal jet exits close to the ventral side of the laryngeal lumen (figure 5*b*), resulting in a glottal jet path nearly parallel to the intralaryngeal supraglottal wall. Such an orientation probably creates a flow pattern inconsistent with an impinging jet model.

Mahrt *et al*. [20] have also investigated the effect of removing the epiglottis as well as parts of the thyroid cartilage. In all three muroid species, we found that the epiglottis is a semicircular structure supporting the supraglottal space on three sides. Surgical removal would either have to be incomplete or would destroy the intralaryngeal supraglottal space, including the ventral pouch. An evaluation of the effect of the surgical interventions performed by Mahrt *et al*. is not possible.

Our attempts to surgically manipulate the ventral pouch confirmed that placement of a lesion is challenging because visualization of the supraglottal and intralaryngeal space is difficult. However, results of our alar lesion experiments suggest that the magnitude of damage to the supraglottal airway, specifically to the ventral pouch, predicts the degree of USV impairment. If the alar edge remained intact, the rat continued to produce USVs. By contrast, destruction of the alar edge abolished USV production. If repair mechanisms are sufficient to restore the alar edge and ventral pouch, the ability to produce USVs may be recovered.

Mahrt *et al*. [20] observed that ultrasonic sounds were rescued after replacing destroyed structures with a small metal plate in the supraglottal space. The fundamental frequency of the reproduced sounds moved relatively slowly up and down. However, the ultrasonic sounds produced with metal plate placement showed some structural differences. Although the spectral contour was similar to natural USVs, experimental sounds were much longer in duration, suggesting that the mechanism can reproduce spectral but not necessarily temporal features, for instance modulation rate of fundamental frequency. We hypothesize that one of the two scenarios may have occurred to restore USVs. First, the ventral pouch remained intact after surgical intervention and the metal plate did not prevent its function. Alternatively, destruction of the supralaryngeal cavity (including the ventral pouch) with subsequent metal plate placement triggered a feedback mechanism (perhaps even a planar impinging jet mechanism) different from the one present with an intact supraglottal space. The difference between natural and experimental sounds highlights the potential importance of motor control for USV production, which was not simulated.

Thus, we conclude that although the study by Mahrt *et al*. [20] confirms earlier findings that the larynx is sufficient to serve as source for ultrasound, the hypothesis that whistles are produced by a high-speed impingement jet onto a planar surface is incongruent with the anatomy of the larynx.

#### 4.1.3. The edge-tone model

We propose an alternative mechanism that considers the ventral pouch central to ultrasonic whistle production mechanism. The edge enforced by the alar cartilage opposite the glottal exit resembles the design of an edge-tone whistle (figure 8*c*). The configuration of an edge-tone whistle has a flat air jet impinging onto a sharp edge of a plate that extends in the same direction as the jet width. If the jet is deflected to flow on one side of the edge, then viscous effects draw air from the other side to become entrained, causing a pressure difference across the two sides of the plate. If the jet flips from one side of the plate to the other, a pressure wave is generated that propagates back to the glottal aperture and slightly deflects its motion (e.g. [52,53]). This deflection grows to form a ‘sinuous’ wave on the jet which maintains the oscillatory deflection of the jet at the alar edge.

In addition to an edge-tone mechanism, we propose that the air jet probably interacts with the resonance of the ventral pouch. The nature of the resonator could be a simple Helmholtz cavity with a single major resonance or a closed pipe-like cavity with a nearly harmonic resonance series [53]. Deflection of the glottal jet at the alar edge is the driving mechanism, wherein sound frequency is determined by the airflow speed exiting the glottis, dimensions of the ventral pouch, and the distance between glottal exit and alar edge (figure 8*c*).

### 4.2. Active control of the ventral pouch geometry and its effect on whistle frequency

We found that a small portion of the thyroarytenoid muscle attaches to the endpoints of the alar cartilage, suggesting the possibility of active control of the position of the alar edge relative to the glottis. Inagi *et al*. [27] provided a description of intrinsic laryngeal musculature of the rat larynx and identified a muscle that inserts into the alar cartilage. The authors suggested that the muscle also attaches to both the arytenoid and cricoid cartilage and therefore labelled it *alar cricoarytenoid muscle*. We were not able to identify the continuation of the muscle beyond the arytenoid cartilage (i.e. further dorsal to the cricoid cartilage), but could only confirm its insertion to the arytenoid and alar cartilage as well as its close association with the main body of the thyroarytenoid muscle. We therefore label the muscle as *alar portion of the thyroarytenoid muscle*.

Contraction of the alar portion of the thyroarytenoid muscle would move the alar edge towards the glottis and reduce the distance between alar and glottal edge. Such movement would also probably affect the cavity size of the ventral pouch. As is well known in flute playing, fundamental frequency is determined not only by airflow velocity but also by the distance between the jet exit (here, the glottis) and the edge (here, the alar edge) (figure 8*c*). Adjusting the distance between glottal and alar edge helps produce optimum conditions for higher frequency notes [52]. The frequency of an edge tone produced by a glottal jet impinging on the alar edge would likely decrease with increasing distance (figure 8*c*) and may break into different modes as distance is further increased [51].

Indeed, rats control vocal fold movements during USV production [15,32,54,55]. Fundamental frequency contour is tightly associated with electromyographic activity of the thyroarytenoid and the cricothyroid muscles [32]. For example, thyroarytenoid muscle activity is higher in amplitude when rats produce 50 kHz compared with 22 kHz calls [32]. Contraction of the thyroarytenoid muscle may have one of the two effects. First, contraction may shorten the vocal fold by pulling the thyroid cartilage towards the arytenoid cartilage. Alternatively, contraction may stiffen the vocal fold body via isometric contraction if the cricothyroid muscle counters the action of the thyroarytenoid muscle to prevent thyroid cartilage movement. Further experimental studies that examine the muscle activity in relation to ventral pouch dynamics are needed to test between the alternative hypotheses. However, electromyographic activity of both muscles in vocalizing rats suggests that neither muscle equally nor synchronously increases, but act in a call-type-specific manner [15,32,54]. High call frequencies could be supported by a shorter glottal length or distance between the glottal and alar edge, and/or by a smaller ventral pouch volume (figure 8). Ultimately, fine control of each variable would determine call-type specificity and define the mechanistic foundation for acoustic variation and diversification among rodents.

## 5. Conclusion

Vocalizations used in social communication are among the most diverse and elaborate displays in the animal kingdom, yet our understanding of the physiological mechanisms driving acoustic divergence remains understudied [56]. Although there has been a long-standing interest in rodent laryngeal anatomy (e.g. [22,23,28,57–59]), a refined morphological treatment has been lacking until now. Laryngeal functional morphology will be critical to inform the taxonomy and evolution of rodents, a group that represents more than 40% of mammalian diversity. While the order contains about 30 higher-level clades [60], USVs have been reported in a relatively select few and is biased towards laboratory strains (e.g. [61–63]). Our finding suggests that the evolution of ultrasonic whistling in rodents for social communication is associated with morphological adaptations of the vocal organ. A broader sampling of rodent laryngeal morphology will not only inform our understanding of the relationship between acoustic and morphological complexity, but also how we interpret molecular genetic mechanisms of laryngeal development and function [28].

Finally, our data highlight the importance of morphological features, either heritable or acquired, as important sources of acoustic variation in USVs. Rodent USVs are widely used as a proxy to evaluate social communication in biomedical research models (e.g. [64,65]). Understanding the mechanistic underpinnings of vocal production will improve our ability to ascribe acoustic differences (e.g. among genetically distinct populations or different experimental treatments) to differences in either morphology or vocal motor control. Such information is critical to how we interpret and gain new insights into this remarkable form of animal communication.

## Supplementary Material

Supplemental Table S1

## Data accessibility

Derived three-dimensional surfaces (STL format) and video animations of all specimens have been archived and are available from Morphobank [66], project no. 2686.
