# Supplementary material for: Laryngeal airway reconstruction indicates that rodent ultrasonic vocalizations are produced by an edge-tone mechanism
Source: R Soc Open Sci. 2017 Nov 1;4(11):170976. doi: 10.1098/rsos.170976 (PMC5717665; doi:10.1098/rsos.170976)
Supplement: Supplemental Table S1 [file rsos170976supp1.docx]

**Laryngeal airway reconstruction indicates rodent ultrasonic vocalizations are produced by an edge tone mechanism**

**Tobias Riede, Heather L. Borgard, Bret Pasch**

**Supplemental Table S1**: Iodine staining protocols

| **Specimen** | **Fixation (d, days)** | **Staining (d, days)** |
| --- | --- | --- |
| Grasshopper mice  (*O. arenicola; O. leucogaster*); A06-01; L05-04 | 10% NBF (2d); 70% ethanol (4d) | 0.4% iodine;  3 and 14 days |
| Grasshopper mouse  (hybrid *O. arenicola x torridus*; *O. torridus*)  AT03-03; T013-01 | 4% paraformaldehyde (initial perfusion); 10% NBF (2d); 70% ethanol (4d) | 1% iodine;  2 x 10 days |
| Four house mice (*Mus musculus*); two males and two females, CD-1 strain | 4% paraformaldehyde (initial perfusion); 10% NBF (2d); 70% ethanol (4d) | 1% iodine;  2 x 10 days |
| One laboratory rat (*Rattus domesticus*); male, Sprague-Dawley strain | 4% paraformaldehyde (initial perfusion); 10% NBF (2d)  70% ethanol (4d) | 1% iodine;  2 x 10 days |
| Three laboratory rats; two males, two females, Sprague-Dawley strain | 10% NBF (2d); 70% ethanol (4d) | 0.4% iodine;  3 and 14 days |
| Four Kangaroo rats (*Dipodomys ordii*); two males and two females, CD-1 strain | 4% paraformaldehyde (initial perfusion); 10% NBF (2d); 70% ethanol (4d) | 1% iodine;  2 x 10 days |
